# Personalized PLGA/BCL Scaffold with Hierarchical Porous Structure Resembling Periosteum‐Bone Complex Enables Efficient Repair of Bone Defect

**DOI:** 10.1002/advs.202401589

**Published:** 2024-07-17

**Authors:** Mengqi Zhang, Zhike Huang, Xun Wang, Xinyu Liu, Wenyi He, Yan Li, Dingcai Wu, Shuyi Wu

**Affiliations:** ^1^ Hospital of Stomatology Guanghua School of Stomatology Guangdong Provincial Key Laboratory of Stomatology Sun Yat‐sen University Guangzhou 510055 P. R. China; ^2^ Medical Research Institute Guangdong Provincial People's Hospital (Guangdong Academy of Medical Sciences) Southern Medical University Guangzhou 510080 P. R. China; ^3^ Key Laboratory for Polymeric Composite and Functional Materials of Ministry of Education School of Chemistry Sun Yat‐sen University Guangzhou 510006 P. R. China

**Keywords:** angiogenesis, bone scaffolds, osteogenesis, personalized preparation

## Abstract

Using bone regeneration scaffolds to repair craniomaxillofacial bone defects is a promising strategy. However, most bone regeneration scaffolds still exist some issues such as a lack of barrier structure, inability to precisely match bone defects, and necessity to incorporate biological components to enhance efficacy. Herein, inspired by a periosteum‐bone complex, a class of multifunctional hierarchical porous poly(lactic‐*co*‐glycolic acid)/baicalein scaffolds is facilely prepared by the union of personalized negative mold technique and phase separation strategy and demonstrated to precisely fit intricate bone defect cavity. The dense up‐surface of the scaffold can prevent soft tissue cell penetration, while the loose bottom‐surface can promote protein adsorption, cell adhesion, and cell infiltration. The interior macropores of the scaffold and the loaded baicalein can synergistically promote cell differentiation, angiogenesis, and osteogenesis. These findings can open an appealing avenue for the development of personalized multifunctional hierarchical materials for bone repair.

## Graphical abstract

By the union of personalized negative mold technique and phase separation strategy, poly(lactic‐*co*‐glycolic acid)/baicalein scaffold is prepared with a hierarchical porous structure resembling a periosteum‐bone complex. The scaffold not only precisely matches the shape of bone defects, but also provides barrier function, protein adsorption, cell‐adhesion promotion, pro‐angiogenesis, and pro‐osteogenesis to protect and regulate the osteogenic microenvironment, resulting in outstanding bone regeneration.

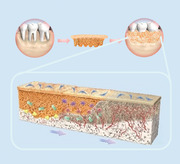

## 1 Introduction

For tissue repair, efficient fabrication of 3D multifunctional biomaterials with tailored sizes, shapes, and structures is highly demanded yet extremely challenging.^[^ ^1^ ^]^ Craniomaxillofacial bone defects, typically exhibiting complex shapes and involving interfaces with different tissues,^[^ ^2^ ^]^ are a formidable issue in the field of tissue repair. As a promising strategy for solving this problem, bone regeneration scaffolds are developed to simulate the barrier function of the periosteum^[^ ^3^ ^]^ and the hierarchical porous structure of bone tissue,^[^ ^4^ ^]^ which can protect and regulate the bone healing processes. The key to this strategy is to enable convenient preparation of personalized bone regeneration scaffolds that meet intricate biological needs.^[^ ^2^ ^]^ On one hand, the scaffolds should mimic the hierarchical porous structure of natural periosteum‐bone complex and possess multiple functionalities to initiate, promote, and sustain bone healing.^[^ ^3^, ^5^ ^]^ On the other hand, the scaffolds that can precisely match the geometric shapes of defect cavities need to be prepared using reliable and efficient methods.^[^ ^2^, ^6^ ^]^

In recent decades, efforts have been made to design and fabricate bone regeneration scaffolds, but some critical aspects still remain unresolved. For example, 3D printing technologies enable the production of scaffolds with specific shapes and porous structures.^[^ ^4^, ^7^ ^]^ But most of these scaffolds exhibit a deficiency in the barrier function of the natural periosteum and cannot prevent soft tissue cell invasion, which can damage the osteogenic microenvironment.^[^ ^8^ ^]^ Their pore walls generally lack internal channel networks formed by transverse secondary pores, thus limiting the nutrient transport inside the scaffolds.^[^ ^9^ ^]^ Meanwhile, the structural design of existing scaffolds often ignores the regulation of porous structure on the early initiation of bone healing biological processes,^[^ ^4^, ^10^ ^]^ potentially resulting in delayed bone healing progress. In addition, bone regeneration scaffolds are frequently prepared via complex fabrication methods to obtain hierarchical porous structures and require the incorporation of cells and/or cytokines to enhance their bioactive functions.^[^ ^4^, ^11^ ^]^ However, these methods are constrained by high costs and complex regulatory requirements, limiting their clinical applications.^[^ ^2^ ^]^ As such, existing bone regeneration scaffolds are still far from ideal 3D bone repair materials. Therefore, how to rationally design and construct multifunctional scaffolds that can simulate the hierarchical porous structure of periosteum‐bone complex and precisely match the geometric shapes of defect cavities is an important scientific and clinical problem that urgently needs to be solved.

**Figure 1:**
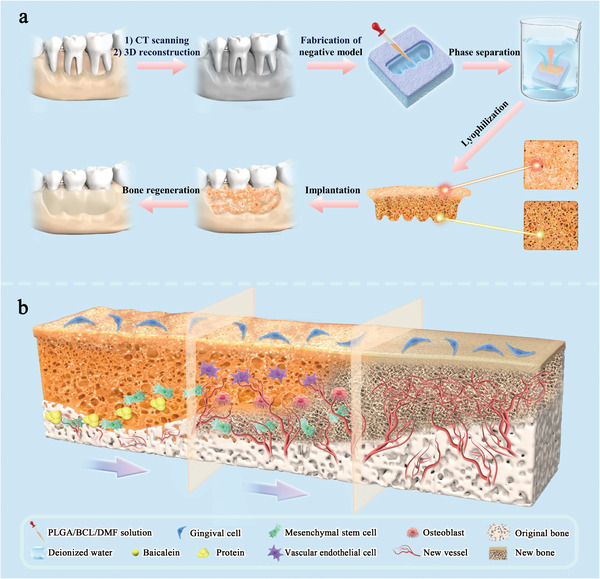
Schematic illustration of personalized fabrication and bone repair processes of PLGA/BCL‐S with hierarchical porous structure. a) The bone defect model is obtained through computer tomography (CT) scanning and subsequent 3D reconstruction. The personalized negative mold of the scaffold is designed and fabricated using CAD/CAM technology. Based on the negative mold, a corresponding scaffold PLGA/BCL‐S, which matches well with the bone defect cavity, is prepared through phase separation and lyophilization. b) The scaffold serves multiple functions: the dense up‐surface prevents the infiltration of soft tissue cells; the smaller macropores on the bottom‐surface facilitate rapid protein adsorption and cell adhesion, while the larger macropores enable cell infiltration into the scaffold; the interior macropores and loaded BCL synergistically promote cell differentiation, angiogenesis, and osteogenesis. The aforementioned functions of the scaffold ultimately result in successful bone regeneration.

Herein, inspired by the hierarchical porous structure of periosteum‐bone complex, we combine the structural plasticity of poly(lactic‐*co*‐glycolic acid) (PLGA) and the pro‐angiogenic and pro‐osteogenic properties of baicalein (BCL) to successfully create a class of multifunctional porous PLGA/BCL scaffold (PLGA/BCL‐S) by the union of personalized negative mold technique and phase separation strategy (**Figure** **1**). We accurately extract the 3D model of bone defect from radiographic data, obtain personalized negative mold using computer‐aided design and manufacturing (CAD/CAM), and then prepare PLGA/BCL‐S through phase separation, followed by lyophilization. The as‐constructed scaffold has a dense up‐surface with macropore sizes ranging from ≈ 1 to 6 µm, effectively establishing a periosteum‐like barrier to prevent the invasion of soft tissue cells into the bone defect area.^[^ ^8^, ^12^ ^]^ The bottom‐surface of the scaffold has unique dual macropores: smaller than 10 µm and larger than 100 µm. These smaller macropores play a role in mediating early biological events such as protein adsorption and cell adhesion during bone healing, while these larger macropores benefit cell migration into the scaffold and provide a foundation for subsequent important processes.^[^ ^13^ ^]^ Resembling the dense structure of cortical bone transitioning to the loose structure of cancellous bone,^[^ ^14^ ^]^ the interior of the scaffold exhibits a hierarchical porous structure from top to bottom. The loose structure contains macropores (ranging from 100 to 300 µm), facilitating the formation of vascular networks and mineralized bone tissue.^[^ ^15^ ^]^ These macropores maintain high connectivity through a large number of connected transverse smaller macropores on the wall, which is conducive to nutrient transport.^[^ ^9^ ^]^ Furthermore, BCL loaded on the scaffold can effectively synergize the aforementioned hierarchical porous structure, endowing the scaffold with excellent pro‐angiogenic and pro‐osteogenic effects.^[^ ^16^ ^]^ Based on the well‐orchestrated structural and functional integration, our hierarchical porous PLGA/BCL‐S exhibits remarkable bone regeneration effect in rats and displays precise matching with large‐sized bone defects in porcine mandible and human maxilla. We believe that our strategy could serve as a novel method for the hierarchical design, personalized preparation, and clinical application of bone repair materials, as well as other tissue repair materials.

## 2 Results and Discussion

### 2.1 Preparation and Characterization of Scaffolds

**Figure 2:**
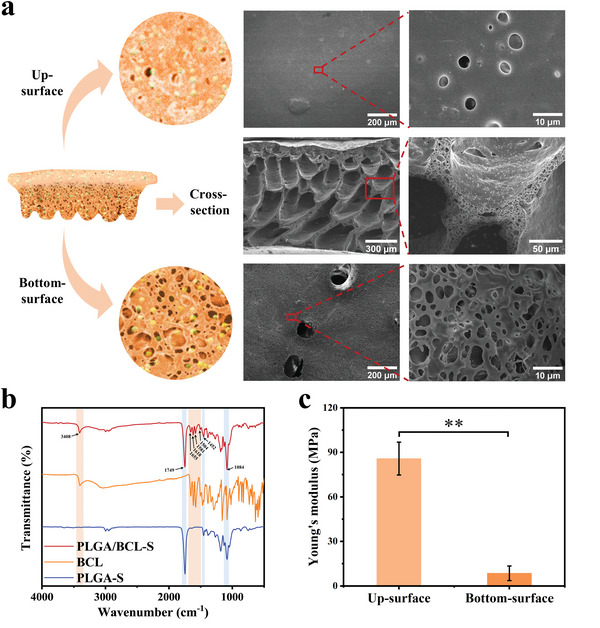
Structural and mechanical characterizations of scaffolds. a) Schematic illustration and SEM images of the hierarchical porous structure of PLGA/BCL‐S. b) FTIR spectra of PLGA‐S, BCL, and PLGA/BCL‐S. c) Young's moduli via atomic force microscopy for the up‐surface and the bottom‐surface of PLGA/BCL‐S in a wet state (n = 3 independent samples; Student's *t* test; ^**^ *p* < 0.01; error bars = standard deviation (SD)).

The preparation procedures of PLGA/BCL‐S include preparation of a cylindrical polytetrafluoroethylene negative mold to form a specific geometric shape, phase separation of PLGA/BCL/N, N‐dimethylformamide (DMF) solution in deionized water to construct an integrated scaffold with hierarchical porous structure, and lyophilization to remove residual solvent and water. Scanning electron microscope (SEM) images (**Figure** **2**a) show that the up‐surface of PLGA/BCL‐S possesses a dense structure with macropore sizes ranging from 1 to 6 µm, which are less than the sizes of fibroblasts (≈10–15 µm^[^ ^17^ ^]^). Thus, the up‐surface mimics the dense structure of the periosteum, effectively acting as a barrier against fibroblasts infiltration during bone regeneration.^[^ ^3^, ^18^ ^]^ The bottom‐surface of PLGA/BCL‐S exhibits a comparably loose structure featuring unique dual macropores: smaller than 10 µm and larger than 100 µm. Smaller macropores are believed to facilitate protein adsorption and enhance stem cell adhesion, while larger macropores could support the migration and differentiation of adhered stem cells within the scaffold.^[^ ^14^, ^19^ ^]^ Notably, the interior of the scaffold resembles the hierarchical structure of natural bone tissue from top to bottom, exhibiting the dense structure and the loose structure.^[^ ^14^ ^]^ These macropores (average pore size of 175 µm) of the loose structure could benefit the formation of vascular networks and mineralized bone tissue.^[^ ^2^ ^]^ Furthermore, the walls of these macropores feature a network of interconnected pores that could facilitate nutrient transport.^[^ ^20^ ^]^ This well‐developed porous structure enables a significant surface area for full interaction with extracellular matrix (ECM),^[^ ^5a^ ^]^ promoting blood vessel ingrowth.^[^ ^19^ ^]^ As shown in Fourier transform infrared (FTIR) spectrum of PLGA/BCL‐S (Figure 2b), C═O stretching peak at 1749 cm^−1^, C─H in‐plane deformation at 1452 cm^−1^, and C─O─C stretching peak at 1084 cm^−1^ are from PLGA.^[^ ^21^ ^]^ The peaks of PLGA/BCL‐S at 1504, 1581, 1618, and 1655 cm^−1^ are ascribed to C═C stretching vibration of the aromatic rings of BCL, and the characteristic peak at 3408 cm^−1^ is ascribed to O─H stretching vibration of BCL.^[^ ^22^ ^]^ The FTIR spectra fully demonstrate that BCL has been successfully loaded in PLGA/BCL‐S without affecting its chemical structure. The average Young's moduli of the dense up‐surface and the loose bottom‐surface of PLGA/BCL‐S in a wet state are 85.8 and 8.5 MPa, respectively (Figure 2c). The harder up‐surface helps maintain the osteogenic space,^[^ ^23^ ^]^ while the softer bottom‐surface facilitates a better fit within the defect cavity. Therefore, we successfully fabricate PLGA/BCL‐S that mimics the hierarchical porous structure of natural periosteal‐bone complex. Moreover, the PLGA scaffold (PLGA‐S) without BCL is also prepared as the control sample using the aforementioned method. The as‐obtained PLGA‐S has a hierarchical porous structure similar to PLGA/BCL‐S (Figure S1, Supporting Information).

### 2.2 Personalized Design of Scaffolds Matching Large‐Sized Defects in Porcine Mandible and Human Maxilla

**Figure 3:**
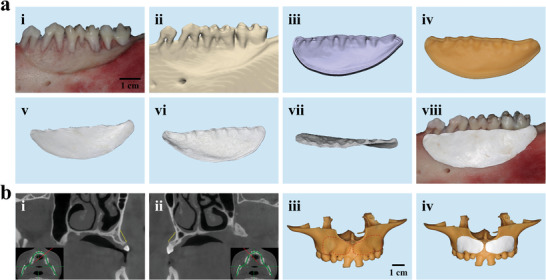
The processes of personalized fabrication of PLGA/BCL‐S matching large‐sized defects in porcine mandible and human maxilla. a) The scaffold for bone defect in a porcine mandible: i) an extensive bone defect created in the posterior region of a porcine mandible; ii) 3D reconstruction of the bone defect based on CT scanning; iii,iv) the negative mold of the target scaffold with a brim‐like structure designed (iii) and manufactured (iv) via CAD/CAM technology; v–vii) digital photos of top view (v), bottom view (vi), and occlusal view (vii) of PLGA/BCL‐S; viii) digital photo of PLGA/BCL‐S placed in bone defect region, exhibiting good matching of the scaffold with the bone defect. b) The scaffolds for bone defects in a patient's maxilla: i,ii) oblique sagittal images of cone beam CT of left (i) and right (ii) maxillary defects (the yellow dashed lines mean the normal positions of the labial bone walls; insets: corresponding cross‐section images; the red lines in the insets represent the positions where oblique sagittal images are captured); iii) digital photo of front view of the resin model of the patient's maxilla (red dashed lines outline the regions of bilateral defects); iv) digital photo of front view exhibiting that PLGA/BCL scaffolds can match well with bilateral bone defects in the resin model.

Craniomaxillofacial bone defects typically have complex shapes and can lead to facial or cranial anomalies, especially in cases involving large‐sized bone defects.^[^ ^2^ ^]^ Hence, it is extremely important to fabricate scaffolds that precisely match both the geometry of defect and the contour of surrounding bone.^[^ ^2^, ^6^ ^]^ As shown in **Figure** **3**a, we create a large‐sized alveolar bone defect in the posterior region of porcine mandible to simulate a clinically unfavorable bone defect, followed by CT scanning and 3D reconstruction. A negative mold of the target scaffold with a brim‐like structure is then designed and manufactured using CAD/CAM technology. According to our aforementioned procedure, the phase separation of PLGA/BCL/DMF solution in deionized water is carried out, followed by lyophilization to obtain the corresponding scaffold. We find that the loose bottom‐surface of the PLGA/BCL‐S product matches well with the irregular morphology of the exposed root and damaged bone, while the dense up‐surface effectively restores the contour of the alveolar bone and completely seals the edge of the defect cavity. To further verify the clinical feasibility of this strategy, we select a clinical case with bilateral maxillary defects caused by congenital permanent teeth loss as a typical example. Based on the same method, we prepare bilateral personalized scaffolds that can precisely match the corresponding defects on both sides (Figure 3b). These scaffolds are expected to effectively restore the labial fullness of alveolar bone and eliminate the necessity for complex bone augmentation surgeries, such as fixing the barrier membrane with pins and extensively expanding the range of mucoperiosteal flap. This approach may conveniently ensure the availability of sufficient bone mass for subsequent dental implantation. Therefore, we successfully achieve personalized customization of scaffolds that accurately fit bone defects by utilizing clinically available equipment and simple preparation processes. This holds great promise for facilitating the clinical translation and application of bone regeneration scaffolds.

### 2.3 Optimization of Baicalein Loading in Scaffolds

**Figure 4:**
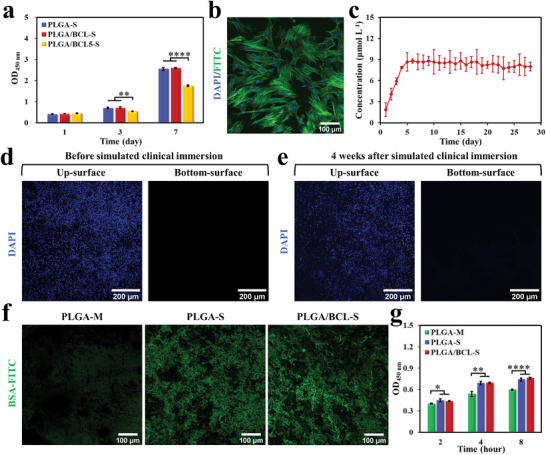
Evaluation of BCL loading proportion, barrier function, protein adsorption, and cell‐adhesion promotion of PLGA/BCL‐S. a) CCK‐8 assay results of BMSCs cultured on the bottom‐surfaces of PLGA‐S, PLGA/BCL‐S, and PLGA/BCL5‐S for 1, 3, and 7 days (n = 3 independent samples; one‐way analysis of variance (ANOVA) followed by least significant difference (LSD) test; ^**^ *p* < 0.01, ^****^ *p* < 0.0001). b) Morphology of BMSCs cultured on the bottom‐surface of PLGA/BCL‐S for 3 days (green for F‐actin and blue for cell nucleus). c) BCL released curve of PLGA/BCL‐S in PBS at different interval times (n = 3 independent samples). d,e) Fluorescence images of L929s cultured on the up‐surface and penetrated cells on the bottom‐surface of PLGA/BCL‐S after 7 days, exhibiting good barrier function of PLGA/BCL‐S against fibroblasts before (d) and after (e) simulated clinical immersion for 4 weeks (blue for cell nucleus). f) Fluorescence images of BSA adsorbed on the bottom‐surfaces of PLGA‐M, PLGA‐S, and PLGA/BCL‐S (green for BSA). g) BMSCs adhered on the bottom‐surfaces of PLGA‐M, PLGA‐S, and PLGA/BCL‐S were detected by CCK‐8 assay after culturing for 2, 4, and 8 hours (n = 3 independent samples; one‐way ANOVA followed by LSD test; ^*^ *p* < 0.05, ^**^ *p* < 0.01, ^****^ *p* < 0.0001). All error bars = SD.

BCL is a flavonoid extracted from the traditional Chinese medicine *Scutellaria baicalensis*. It is a 5,6,7‐trihydroxy flavone with the molecular formula C_15_H_10_O_5_. It contains the hydroxyl group, the carbonyl group, and the C═C bond group. These active groups are responsible for the multifunctional activity of baicalein, such as promoting angiogenesis and osteogenesis. But its activity is closely related to concentration.^[^ ^24^ ^]^ Hence, it is essential to determine the appropriate loading proportion of BCL in the PLGA/BCL‐S to ensure that its safe effective concentration and sustained release duration align with the requirements for bone regeneration. Bone marrow mesenchymal stem cells (BMSCs) are seeded onto scaffolds, which exhibit different mass ratios of BCL to PLGA, including 0‰ for PLGA‐S, 2.5‰ for PLGA/BCL‐S, and 5‰ for PLGA/BCL5‐S. As illustrated in **Figure** **4**a, the cell viability of PLGA‐S, PLGA/BCL‐S, and PLGA/BCL5‐S groups significantly increase over time and there is no significant difference between PLGA‐S and PLGA/BCL‐S groups. However, cell viability in PLGA/BCL5‐S group shows a significant decrease on the 3rd and 7th days compared to PLGA‐S group. This reduction may be attributed to the BCL loading concentration surpassing its safe threshold, which affects cell proliferation.^[^ ^25^ ^]^ Fluorescence staining results reveal that BMSCs in both PLGA‐S and PLGA/BCL‐S groups are fully extended, with the cytoskeleton arranged in a filamentous pattern (Figure 4b; Figure S2, Supporting Information). To assess the release behavior of BCL in the as‐constructed PLGA/BCL‐S, samples are immersed in phosphate buffer solution (PBS). At each time point, 100 µL solution is extracted to quantify the concentrations of released BCL, after which an equal amount of fresh PBS is replenished. As shown in Figure 4c, the concentrations of BCL are slowly increased in the first 5 days and sustained in ≈ 9 µmol L^−1^ in the following 23 days. The results demonstrate that our PLGA/BCL‐S can achieve sustained release of BCL, which may be due to the low water solubility of BCL in the suitable porous structure of the scaffold (*S*_BET_ of PLGA/BCL‐S was 14 m^2^ g^−1^; Figure S3, Supporting Information).^[^ ^26^ ^]^ Therefore, PLGA/BCL‐S has excellent cytocompatibility and can stably release BCL.

### 2.4 Barrier Function of Scaffolds

When a bone defect occurs, the bone tissue and its surrounding soft tissues engage in a competition to fill the defect cavity. Given their faster growth rates, soft tissues often prevail in occupying the bone defect area, ultimately compromising the effectiveness of bone regeneration.^[^ ^27^ ^]^ Therefore, to ensure a successful outcome, it is important for bone repair biomaterials to possess a durable barrier function against soft tissue cells invasion.^[^ ^23^, ^28^ ^]^ In order to evaluate the barrier function, mouse fibroblasts (L929s) are seeded on the up‐surfaces of the samples, and the cells that penetrate and reach the bottom‐surfaces are detected after 3 and 7 days. The results show that almost no cells can reach the bottom‐surfaces of the samples, although fibroblasts can adhere and proliferate well on the up‐surfaces (Figure 4d; Figures S4 and S5, Supporting Information). We further conduct simulating clinical immersion of PLGA‐S and PLGA/BCL‐S for 4 weeks to test the long‐term effectiveness in barrier function. As expected, the dense up‐surfaces of PLGA‐S and PLGA/BCL‐S still can prevent cells penetration (Figure 4e; Figure S7, Supporting Information). The excellent barrier functions of these two scaffolds are also supported by the cross‐section images (Figures S6 and S8, Supporting Information). These results demonstrate that the dense up‐surfaces of PLGA‐S and PLGA/BCL‐S can achieve long‐term protection of the osteogenic microenvironment.

### 2.5 Protein Adsorption and Cell‐Adhesion Promotion Properties of Scaffolds

Favorable interactions between a biomaterial and the host can be enhanced by adsorbing proteins, which can further mediate the early adhesion of cells.^[^ ^2^, ^5^ ^]^ To evaluate the protein adsorption capacity of the porous structures of PLGA‐S and PLGA/BCL‐S, we prepare a relatively non‐porous PLGA membrane (PLGA‐M; Figure S9, Supporting Information) as the control sample for comparative analysis. The samples are soaked in a solution of bovine serum albumin labeled with fluorescein isothiocyanate (BSA‐FITC), and the amount of protein adsorbed on the bottom‐surfaces of the samples is measured after 2 hours. The fluorescence images and the corresponding quantitative analyses demonstrate that the bottom‐surfaces of both PLGA‐S and PLGA/BCL‐S exhibit more BSA adsorption compared to PLGA‐M (Figure 4f; Figure S10, Supporting Information). We speculate that the macropores ranging from 1–5 µm on the bottom‐surfaces of the scaffolds facilitate protein adsorption,^[^ ^5b^ ^]^ thereby enabling “self‐modification” of the scaffolds. We further examine the effects of scaffolds on cell adhesion. Cell Counting Kit‐8 (CCK‐8) results show that significantly more BMSCs adhere to the bottom‐surfaces of PLGA‐S and PLGA/BCL‐S after 4 and 8 hours compared to PLGA‐M (Figure 4g). These enhanced cell adhesions may be attributed to the scaffolds’ well‐designed macropore structures and their excellent protein adsorption capabilities.^[^ ^2^, ^5^ ^]^ Based on the aforementioned properties, the loose bottom‐surfaces of our scaffolds are expected to rapidly interact with surrounding tissues and promptly initiate bone healing responses.

### 2.6 In Vitro Pro‐Angiogenic and Pro‐Osteogenic Properties of Scaffolds

**Figure 5:**
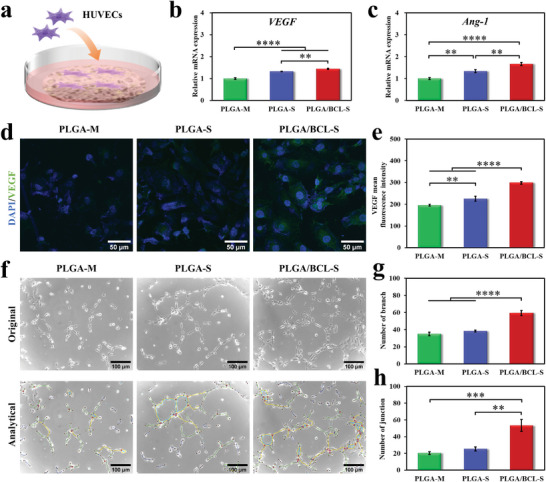
In vitro pro‐angiogenic property of PLGA/BCL‐S. a) Schematic diagram of evaluating the pro‐angiogenic property of scaffold via seeding HUVECs on the bottom‐surface. b,c) RT‐qPCR results of expression levels of angiogenic‐related genes including *VEGF* (b) and *Ang‐1* (c) after culturing cells on the bottom‐surfaces of samples for 3 days (n = 3 independent samples; one‐way ANOVA followed by LSD test; ^**^ *p* < 0.01, ^****^ *p* < 0.0001). d,e) Immunofluorescence staining (d) and semi‐quantitative statistical analyses of fluorescence intensities (e) of VEGF after culturing cells on the bottom‐surfaces of samples for 3 days (green for VEGF and blue for cell nucleus; n = 3 independent samples; one‐way ANOVA followed by LSD test; ^**^ *p* < 0.01, ^****^ *p* < 0.0001). f) Tubule‐formation assay after culturing cells in conditioned culture mediums of samples for 4 hours. In the analytical images, red circle means junction, dark blue dot denotes node, light blue circle suggests mesh, yellow circle signifies master segment, blue line shows extremity, and green line represents branch. g,h) quantitative statistical analyses of branch numbers (g) and junction numbers (h) of tubule‐formation assay (n = 3 independent samples; one‐way ANOVA followed by LSD test; **p < 0.01, ^***^ *p* < 0.001, ^****^ *p* < 0.0001). All error bars = SD.

Despite the abilities of our scaffolds to promote cell adhesion, it is still essential for the scaffolds to provide a good microenvironment to induce differentiation of bone tissue cells.^[^ ^5^, ^19^ ^]^ As new blood vessels transport oxygen and nutrients to the highly metabolically active regenerating tissues, angiogenesis is an important component of bone repair.^[^ ^14^, ^20^ ^]^ To evaluate the pro‐angiogenic properties of our scaffolds, human umbilical vein endothelial cells (HUVECs) are seeded on the bottom‐surfaces of PLGA‐M, PLGA‐S, and PLGA/BCL‐S (**Figure** **5**a). Quantitative reverse transcription polymerase chain reaction (RT‐qPCR) results demonstrate a significant upregulation of angiogenesis‐related genes including *vascular endothelial growth factor* (*VEGF*) and *angiopoietin‐1* (*Ang‐1*) in PLGA‐S and PLGA/BCL‐S groups compared to PLGA‐M group. Remarkably, the highest expressions of both genes are clearly observed in PLGA/BCL‐S group (Figure 5b,c). The results of immunofluorescence staining and semi‐quantitative statistical analyses of VEGF display a consistent trend with the above findings (Figure 5d,e). Moreover, tubule‐formation assay reveals that HUVECs cultured in the conditioned culture medium of PLGA/BCL‐S exhibit an enhanced capacity to form vascular‐like structures after 4 hours of incubation, featuring more branches and junctions compared to PLGA‐M and PLGA‐S groups (Figure 5f–h). Taken together, the rationality of porous structure and the effective regulation of BCL endow PLGA/BCL‐S with favorable pro‐angiogenic property.

**Figure 6:**
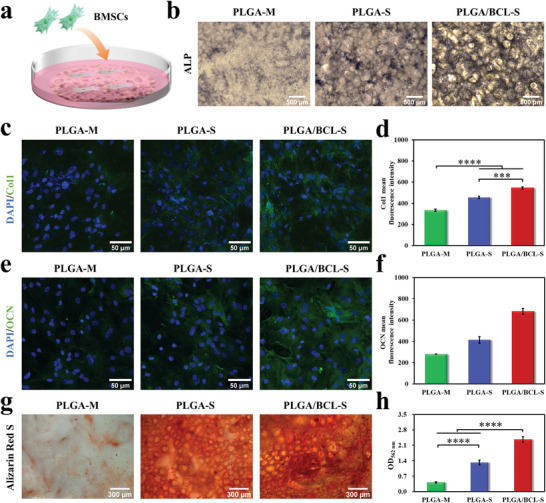
In vitro pro‐osteogenic property of PLGA/BCL‐S. a) Schematic diagram of evaluating pro‐osteogenic property of scaffold by seeding BMSCs on the bottom‐surface. b) ALP staining images of BMSCs cultured in osteogenic induction medium for 7 days. c,d) Col1 immunofluorescence staining (c) and semi‐quantitative statistical analyses of fluorescence intensity (d) after culturing cells in osteogenic induction medium for 7 days (green for Col1 and blue for cell nucleus; n = 3 independent samples; one‐way ANOVA followed by LSD test; ^***^ *p* < 0.001, ^****^ *p* < 0.0001). e,f) OCN immunofluorescence staining images (e) and semi‐quantitative statistical analyses of fluorescence intensity (f) after culturing cells in osteogenic induction medium for 14 days (green for OCN and blue for cell nucleus; n = 3 independent samples; one‐way ANOVA followed by LSD test; ^**^ *p* < 0.01, ^***^ *p* < 0.001, ^****^ *p* < 0.0001). g,h) Alizarin Red S staining images (g) and semi‐quantitative statistical analyses (h) of calcium deposit after culturing cells in osteogenic induction medium for 21 days (n = 3 independent samples; one‐way ANOVA followed by LSD test; ^****^ *p* < 0.0001). All error bars = SD.

The pro‐osteogenic ability which is considered to be essential for bone regeneration is assessed.^[^ ^19^, ^29^ ^]^ BMSCs are seeded on the bottom‐surfaces of PLGA‐M, PLGA‐S, and PLGA/BCL‐S (**Figure** **6**a). The pro‐osteogenic properties of the scaffolds are comprehensively assessed by detecting early osteogenic markers including alkaline phosphatase (ALP) and type I collagen (Col1) as well as late osteogenic markers including osteocalcin (OCN) and calcium nodule formation. In the results, a clear trend is consistently observed: PLGA/BCL‐S group exhibits superior pro‐osteogenic performance, followed by PLGA‐S group, while PLGA‐M group performs relatively poor (Figure 6b–h).

In light of the aforementioned results, we believe that PLGA/BCL‐S exerts its modulatory effects on the microenvironment by utilizing its hierarchical porous structure and sustained release of BCL. On one hand, the porous structure parameters of the scaffold, such as pore size, porosity, and interconnectivity, can impact the critical steps of bone regeneration.^[^ ^5a^ ^]^ Different levels of porous structures serve distinct biological functions. For instance, macropores ranging from 100–200 µm promote the generation of mineralized bone and small blood vessels, whereas macropores exceeding 200 µm facilitate the development of large blood vessels with deep penetration depth.^[^ ^30^ ^]^ Moreover, the high porosity provides a large surface area that facilitates the interaction between the scaffold and ECM.^[^ ^5a^ ^]^ The interconnectivity of the scaffold benefits for cell infiltration, nutrient transport, and establishment of vascular networks.^[^ ^20^, ^31^ ^]^ On the other hand, BCL loaded in PLGA/BCL‐S can promote angiogenesis and osteogenesis through various molecular mechanisms. For instance, BCL induces the formation of new blood vessels by inhibiting hypoxia‐inducible factor‐1α‐specific prolyl‐4 hydroxylases,^[^ ^16^, ^32^ ^]^ and enhances the osteogenic differentiation of pre‐osteoblasts via the mammalian target of rapamycin complex 1 pathway.^[^ ^33^ ^]^ Therefore, by coupling the hierarchical porous structure and the function of BCL, PLGA/BCL‐S can effectively achieve dual regulations of angiogenesis and osteogenesis in the bone microenvironment, making it a good scaffold candidate for bone regeneration.

### 2.7 In Vivo Pro‐Angiogenic and Pro‐Osteogenic Properties of Scaffolds

**Figure 7:**
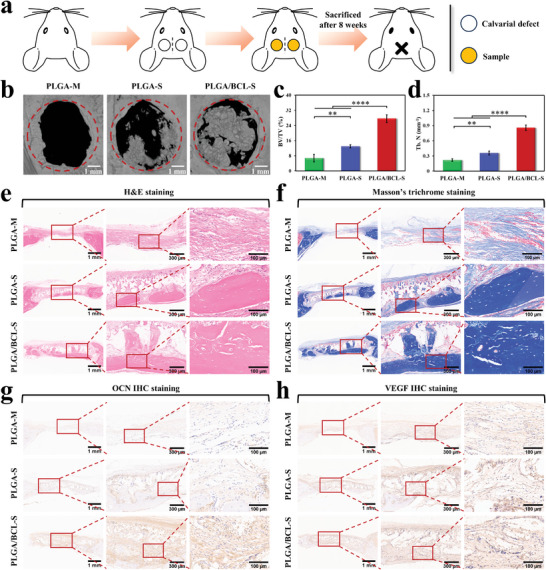
In vivo pro‐angiogenic and pro‐osteogenic properties of PLGA/BCL‐S. a) Schematic diagram showing the process of calvarial defect model in rat to evaluate pro‐angiogenic and pro‐osteogenic properties of our scaffold in vivo. b) Representative images of micro‐CT 3D reconstructions of new bone inside bone defect regions after 8 weeks of implantation. c,d) Quantitative analyses of bone parameters including BV/TV (c) and Tb. N (d) of new bone after 8 weeks of implantation (n = 4 independent samples; one‐way ANOVA followed by LSD test; ^**^ *p* < 0.01, ^****^ *p* < 0.0001; error bars = SD). e,f) H&E staining (e) and Masson's trichrome staining (f) of calvarial decalcified sections after 8 weeks of implantation. g,h) OCN (g) and VEGF (h) IHC staining images of calvarial decalcified sections after 8 weeks of implantation.

The outstanding performances of PLGA/BCL‐S in vitro prompt us to further investigate its capabilities in promoting angiogenesis and osteogenesis in vivo. Two critical‐sized circular bone defects (5.5 mm diameter) are created on both sides of the rat skull, and then implanted with PLGA‐M, PLGA‐S or PLGA/BCL‐S (**Figure** **7**a). To prevent invasion of soft tissue cells into the bone defect area, a brim‐like structure with a slightly wider up‐surface than the defect area is designed, and custom‐made scaffolds matching the sizes of the bone defects are utilized (Figure S11, Supporting Information). After 8 weeks of implantation, 3D reconstruction of micro‐computed tomography (micro‐CT) data clearly reveals distinct differences among groups. In PLGA‐M group, a limited amount of new bone formation is observed only at the edge of the defect, whereas PLGA‐S group exhibits a significant increase in new bone formation. In stark contrast, PLGA/BCL‐S group displays substantial new bone formation both at the edge and center of the defect (Figure 7b). These findings are further supported by quantitative analyses, including measurements of bone volume fraction (BV/TV; Figure 7c) and trabecular number (Tb. N; Figure 7d) of regenerated bone.

The results of hematoxylin and eosin (H&E) staining (Figure 7e) and Masson's trichrome staining (Figure 7f) demonstrate that the newly formed tissues exhibit sufficient thicknesses in both PLGA‐S and PLGA/BCL‐S groups, with no significant difference from the thickness of the surrounding normal tissue. In sharp contrast, the thickness of newly formed tissue in PLGA‐M group is remarkably thin. This difference can be attributed to the 3D structure of PLGA‐S and PLGA/BCL‐S and the appropriate degradation rate of PLGA (complete degradation takes more than 6 months),^[^ ^34^ ^]^ resulting in effective and durable space maintenance.^[^ ^35^ ^]^ The PLGA/BCL‐S group shows a significant presence of mature bone tissue, followed by PLGA‐S group, while PLGA‐M group shows the least amount of mature bone tissue. OCN and VEGF immunohistochemical (IHC) staining and CD31 immunofluorescence staining results display a similar trend to the above findings (Figure 7g,h; Figure S12, Supporting Information). These results can be attributed to the hierarchical porous structure of PLGA/BCL‐S and the multifunctionality of BCL, which can protect and regulate the bone microenvironment. Therefore, PLGA/BCL‐S can promote angiogenesis and osteogenesis in vivo, exerting a beneficial effect on bone repair.

## 3 Conclusion

In conclusion, we have successfully developed a class of periosteum‐bone complex‐inspired porous PLGA/BCL‐S by the union of personalized negative mold technique and phase separation strategy, which facilely achieves scaffold customization to precisely fit the bone defect cavity. The up‐surface of the as‐constructed PLGA/BCL‐S exhibits a periosteum‐like dense structure, which can effectively inhibit fibroblast penetration; the bottom‐surface features unique dual pores that can enhance protein adsorption, cell adhesion, and cell infiltration; the interior porous structure mimics the hierarchical structure of natural bone from cortical to cancellous bone, synergistically promoting angiogenesis and osteogenesis with BCL. With these important advantages, our new scaffold can protect and regulate the osteogenic microenvironment, leading to outstanding repair of bone defects in vivo. We hope that our work would provide a valuable direction for the development of personalized multifunctional bone repair materials with a hierarchical porous structure as well as a novel platform for the design of tissue repair materials in other applications.

## Conflict of Interest

The authors declare no conflict of interest.

## Author Contributions

S.W., D.W., and Y.L. supervised the project. M.Z. and Z.H. conceived the underlying idea. M.Z., Z.H., X.W., X.L., and W.H. designed the research and carried out the experiment. M.Z. and Z.H. performed the statistical analysis. M.Z., Z.H., Y.L., D.W., and S.W. wrote the manuscript. All the authors contributed to discussing and revising the manuscript.

## Supporting information

Supporting Information

## Data Availability

The data that support the findings of this study are available in the supplementary material of this article.
