# Supplementary material for: Personalized PLGA/BCL Scaffold with Hierarchical Porous Structure Resembling Periosteum‐Bone Complex Enables Efficient Repair of Bone Defect
Source: Adv Sci (Weinh). 2024 Jul 17;11(35):2401589. doi: 10.1002/advs.202401589 (PMC11425253; doi:10.1002/advs.202401589)
Supplement: Supplementary file 1 — Supporting Information [file ADVS-11-2401589-s001.docx]

**Supplementary Information**

**Personalized PLGA/BCL Scaffold with Hierarchical Porous Structure Resembling Periosteum‐Bone Complex Enables Efficient Repair of Bone Defect**

*Mengqi Zhang, Zhike Huang, Xun Wang, Xinyu Liu, Wenyi He, Yan Li ^*^, Dingcai Wu ^*^, Shuyi Wu^*^*

M. Zhang, X. Wang, X. Liu, Y. Li, S.Wu

Hospital of Stomatology,

Guanghua School of Stomatology,

Guangdong Provincial Key Laboratory of Stomatology,

Sun Yat-sen University,

Guangzhou 510055, P. R. China.

E-mail: liy8@mail.sysu.edu.cn

E-mail: wushuyi@mail.sysu.edu.cn

Z. Huang

Medical Research Institute,

Guangdong Provincial People's Hospital (Guangdong Academy of Medical Sciences), Southern Medical University,

Guangzhou 510080, P. R. China.

W. He, D. Wu.

Key Laboratory for Polymeric Composite and Functional Materials of Ministry of Education,

School of Chemistry,

Sun Yat-sen University,

Guangzhou 510006, P. R. China.

E-mail: wudc@mail.sysu.edu.cn

**Materials and methods**

**Preparation of poly(lactic-*co*-glycolic acid) scaffold (PLGA-S)**

300 mg PLGA (Daigang, China; LA/GA = 75/25, *M_w_* ≈ 300 kDa) was dissolved in 3 mL N, N-dimethylformamide (DMF; Macklin, China) and stirred for 30 minutes at 55 °C to obtain 10% w/v PLGA/DMF solution. The PLGA/DMF solution was then added dropwise into the cylindrical polytetrafluoroethylene (PTFE) negative mold and soaked in 3 L deionized water for 3 hours at 4 ℃. Samples were completely frozen in liquid nitrogen and lyophilized for 1 day to obtain the PLGA-S products.

**Preparation of PLGA/****baicalein scaffold (PLGA/BCL-S)**

25 mg BCL (Aladdin, China) was dissolved in 1 mL DMF at 20 °C to obtain 2.5% w/v BCL/DMF solution. 30 μL or 60 μL BCL/DMF solution was then added to 3 mL 10% w/v PLGA/DMF solution. The mixture was stirred for 10 minutes at 20 °C and sonicated for 30 minutes at 20 °C, yielding PLGA/BCL/DMF solution with 2.5‰ or 5‰ mass ratio of BCL to PLGA. PLGA/BCL/DMF solutions were added dropwise into cylindrical PTFE negative molds and soaked in 3 L deionized water for 3 hours at 4 ℃. Samples were completely frozen in liquid nitrogen and lyophilized for 1 day, resulting in PLGA/BCL-S or PLGA/BCL5-S products.

**Preparation of PLGA membrane (PLGA-M)**

10% w/v PLGA/DMF solution was added dropwise into a PTFE mold and air-dried in blast drying oven for 1 day at 55 ℃, yielding PLGA-M products.

**Structural and mechanical characterizations**

Morphologies of samples were observed by scanning electron microscope (SEM; S-4800, Hitachi, Japan). Before investigating the cross-sectional morphologies, samples were completely frozen in liquid nitrogen and crushed. N_2_ adsorption measurement was conducted on an autosorb iQ3 (Quantachrome instruments, USA) at 77 K. The Brunauer-Emmett-Teller (BET) surface area and pore size distribution were calculated based on BET theory and original density function theory (DFT), respectively. Fourier transform infrared (FTIR) spectra of samples were recorded by FTIR spectroscopy (Nicolet 6700, Thermo Scientific, America). After immersing PLGA/BCL-S in phosphate buffer solution (PBS) for 24 hours at 37 °C, Young's moduli of its up-surface and bottom-surface were investigated using atomic force microscopy (Dimension FastScan, Bruker, Germany).

**Personalized fabrication of scaffolds matching large-sized defects in porcine mandible and human maxilla**

A large-sized bone defect was created in the posterior region of a porcine mandible. Computer tomography (CT) scanning of the bone defect was performed using Dual Source CT (Force, Siemens, Germany) and 3D reconstruction was generated using Mimic 20.0 software (Materialise, Belgium). The negative mold of target scaffold was designed using 3-Matic 15.0 software (Materialise, Belgium) and printed with a 3D printer (EvoDent S300, UnionTech, China). PLGA/BCL-S was then prepared according to the aforementioned method and placed in the bone defect to check its fitness.

We further selected a clinical case featuring bilateral maxillary defects caused by congenital permanent teeth loss. The procedures were performed under the permission of the Ethics Committee of Hospital of Stomatology, Sun Yat-sen University (No. KQEC-2024-11-01). 3D data of the jaws were collected using a Cone beam CT scanner (NewTom VGi, NewTom, Italy). The resin model of patient’s maxilla was printed with a 3D printer (EvoDent S300, UnionTech, China). The negative molds of the desired scaffolds were designed using 3-Matic 15.0 software (Materialise, Belgium) and printed through the 3D printer. Scaffolds were subsequently prepared following the above method and placed in the resin model to assess their fitness.

**Cytocompatibility evaluation**

The cytocompatibility of PLGA-S, PLGA/BCL-S and PLGA/BCL5-S was tested via Cell Counting Kit-8 (CCK-8) (Dojindo, Tokyo, Japan). Briefly, bone marrow mesenchymal stem cells (BMSCs) were seeded on the bottom-surfaces of scaffolds. On the 1^st^, 3^rd^ and 7^th^ days of culture, 200 μL of 10% CCK-8 reagent was added to each well. After 1 hour of incubation, the optical density (OD) of the supernatant was measured by a microplate reader (Epoch 2, BioTek, USA) at 450 nm. To observe the effect of scaffolds on cell morphology, BMSCs were seeded on the bottom-surfaces of PLGA-S and PLGA/BCL-S. After 3 days of culture, cells were fixed with 4% paraformaldehyde (Biosharp, China) for 30 minutes and permeabilized with 0.5% Triton X-100/PBS solution for 10 minutes. Cells were stained with 4’,6-diamidino-2-phenylindole (DAPI; Beyotime, China) and ActinTracker Green (Beyotime, China). Finally, cell morphologies were observed using confocal laser scanning microscope (CLSM; FV3000, Olympus, Japan).

**In vitro BCL release**

PLGA/BCL-S (≈ 50 mg) was immersed into 500 μL PBS and placed into an incubator at 37 °C in the dark. 100 μL immersion solution was withdrawn at predetermined time points, followed by supplementing 100 μL fresh PBS. To determine the concentration of BCL, a series of BCL/DMF/PBS solutions with known concentrations were prepared and their absorbance at 272 nm were measured by a spectrophotometer (NanoDrop One, Thermo Scientific, America) to produce a standard curve. Subsequently, the concentrations of BCL in the immersion solution were quantified by measuring their absorbance and referring to the standard curve.

**Evaluation of protein adsorption property**

PLGA-M, PLGA-S and PLGA/BCL-S were immersed in 20 μL mL^-1^ bovine serum albumin labeled with fluorescein isothiocyanate (BSA-FITC)/PBS solution, incubated in the dark for 4 hours at 37 ℃, and washed with PBS for 15 minutes. The protein adsorbed on the bottom-surface of each sample was observed by CLSM (FV3000, Olympus, Japan).

**Evaluation of cell-adhesion promotion property**

BMSCs were seeded on the bottom-surfaces of PLGA-M, PLGA-S and PLGA/BCL-S. After 2, 4, and 8 hours of incubation, each sample was transferred to a new well. 200 μL of 10% CCK-8 reagent was added to each well and incubated for 1 hour. The OD of the supernatant at 450 nm was detected using a microplate reader (Epoch 2, BioTek, America).

**Evaluation of barrier function**

PLGA-M, PLGA-S, and PLGA/BCL-S were stored in sterilized PBS for different periods (0 and 4 weeks). At each time point, 20 μL mouse fibroblasts (L929s) suspension was seeded on up-surfaces of samples. After 3 and 7 days of culture, each sample was transferred to a new well. Cells were fixed with 4% paraformaldehyde for 30 minutes and stained with DAPI for 5 minutes. Cells on the up-surfaces and bottom-surfaces were observed using CLSM (FV3000, Olympus, Japan). The scanned images were 3D reconstructed to obtain cross-section images.

**In vitro** **evaluation of pro-angiogenic properties**

Human umbilical vein endothelial cells (HUVECs) were seeded on the bottom-surfaces of PLGA-M, PLGA-S, and PLGA/BCL-S and cultured using Endothelial Cell Medium (ScienCell, America). On the 3^rd^ day, the expression levels of angiogenesis-related genes including *vascular endothelial growth factor* (*VEGF*) and *angiopoietin-1(Ang-1)* were estimated by quantitative reverse transcription polymerase chain reaction (RT-qPCR). Total RNA was isolated from HUVECs using an RNA-Quick Purification Kit (Yishan, China). The cDNA was synthesized using PrimeScript^TM^ RT Reagent Kit (Takara, China). RT-qPCR was performed on a Real-Time PCR system (Roche, LightCycler 96, Switzerland). The expression of VEGF was detected using immunofluorescence technology. Specifically, cells were fixed with 4% paraformaldehyde for 30 minutes, permeabilized with 0.1% Triton X-100/PBS for 15 minutes, and blocked with 3% BSA/PBS for 1 hour at 25 ℃. The primary antibody of VEGF (1:200; Abclonal, China) was added and incubated overnight at 4 °C. The FITC-labeled secondary antibody (1:150; EMAR, China) was added and incubated at 37 °C for 1 hour. Cell nuclei were stained with DAPI for 5 minutes. VEGF expression was observed using CLSM (FV3000, Olympus, Japan). The pro-angiogenic property of PLGA/BCL-S was further evaluated via tubule-formation assay. PLGA-M, PLGA-S, and PLGA/BCL-S were soaked in Endothelial Cell Medium and the conditioned culture medium was collected after 24 hours. HUVECs were resuspended using the conditioned culture medium and seeded on Matrigel (Corning, America) for 4 hours. Tubule formation was observed using a microscope (Zeiss Axio, Leica, Germany). The images were analyzed by Image J software (v1.6.0, National Institute of Health, Bethesda, USA).

**In vitro evaluation of pro-osteogenic properties**

BMSCs were seeded on the bottom-surfaces of PLGA-M, PLGA-S, and PLGA/BCL-S. On the 7^th^ day, samples were fixed with 4% paraformaldehyde for 30 minutes and stained using BCIP/NBT alkaline phosphatase (ALP) Color Development Kit (Beyotime, China). The expression of ALP was observed by stereomicroscope (MZ10F, Leica, Germany). On the 7^th^ and 14^th^ days, the expressions of type I collagen and osteocalcin (OCN) of BMSCs were detected by immunofluorescence technology as described previously. On the 21^st^ day, samples were fixed with 4% paraformaldehyde for 30 minutes and stained with Alizarin Red solution (Cyagen, China). Calcium nodules were observed using stereomicroscope (MZ10F, Leica, Germany). After cutting the samples into small pieces, 1.5 mL of hexadecylpyridinium chloride monohydrate was added to each well and shaken by an ultrasonic cell crusher (Q700, Qsonica, USA) for 30 minutes until the calcium nodules were completely dissolved. 200 μL of supernatant was then collected and the absorbance was measured at 562 nm using a microplate reader (Epoch 2, BioTek, USA).

**In vivo evaluation of pro-angiogenic and pro-osteogenic properties**

All animal experiments in this study were approved by the Institutional Animal Care and Use Committee of Sun Yat-sen University (No. SYSU-IACUC-2023-000165). After intraperitoneal anesthesia with Zoletil 50 (Virbac, France), critical-sized circular bone defects (5.5 mm diameter) were created on both sides of the parietal bone of male Sprague-Dawley (SD) rats (6-8 weeks old, SPF) using a trephine bur (öko Dent, Germany). The defects were implanted with PLGA-M, PLGA-S or PLGA/BCL-S.

After 8 weeks, all rats were sacrificed and calvariums containing the defect regions were removed and immersed into 4% paraformaldehyde for 48 hours. Micro-computed tomography scanning of the bone defects was performed using SkyScan 1276 system (Bruker, Germany) and 3D reconstructions were generated using CTvox software (v.3.3.0, Bruker, Germany). Quantitative analyses of bone volume fraction and trabecular number were conducted. The fixed specimens were subsequently immersed in 10% ethylenediaminetetraacetic acid decalcification solution (Servicebio, China) until complete decalcification was achieved. The specimens were dehydrated using an ascending ethanol gradient, embedded in paraffin, and coronally sectioned into 4-µm-thick slices. Sections were stained with hematoxylin and eosin, Masson's trichrome, immunohistochemistry (IHC) of VEGF and OCN, and immunofluorescence (IF) of CD31. For IHC staining, the primary antibodies were rabbit anti-rat VEGF (1:100; Affinity, China) and rabbit anti-rat OCN (1:100; Affinity, China). The secondary antibody was horseradish peroxidase-labeled goat anti-rabbit lgG (1:200; Servicebio, China). Observation was conducted using a slide scanner (Aperio AT2, Leica, Germany). For IF staining, the primary antibodies was rabbit anti-rat CD31 (1:1000; Abcam, UK). The secondary antibody was Cy3-conjugated goat anti-rabbit lgG (1:200; Abcam, UK). Observation was conducted using a CLSM (FV3000, Olympus, Japan).

**Statistical analysis**

All statistical analyses were carried out by SPSS software (IBM Corp, America). The data were expressed as the mean values ± standard deviation (SD) from at least three parallel experiments. For data with normal distribution and homogeneity of variance, statistical differences between groups were determined by Student's *t* test, one-way analysis of variance (ANOVA) followed by least significant difference (LSD) test. Data with abnormal distribution or heterogeneity of variance were analyzed by Kruskal-Wallis’s nonparametric test. *p* < 0.05 was considered statistically significant.


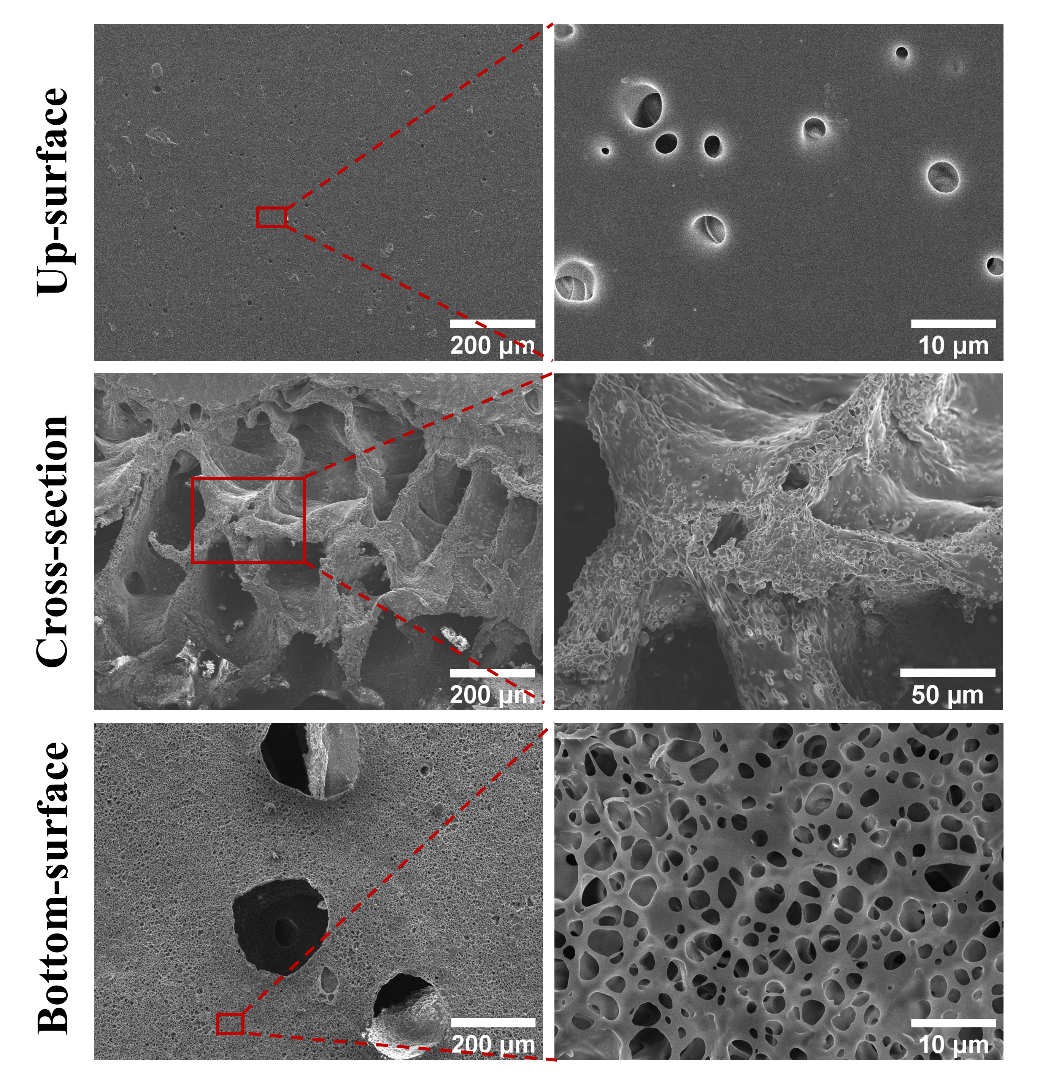


**Figure S1.** SEM images of hierarchical porous structure of PLGA-S.


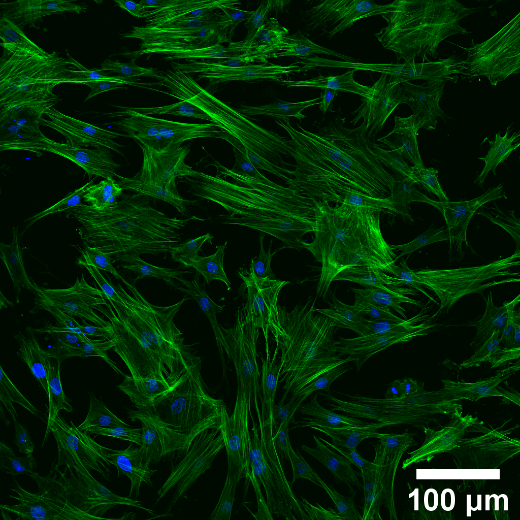


**Figure S2.** Morphology of BMSCs cultured on the bottom-surface of PLGA-S for 3 days (green for F-actin and blue for cell nucleus).





**Figure S3**. N_2_ adsorption–desorption isotherm of PLGA/BCL-S.


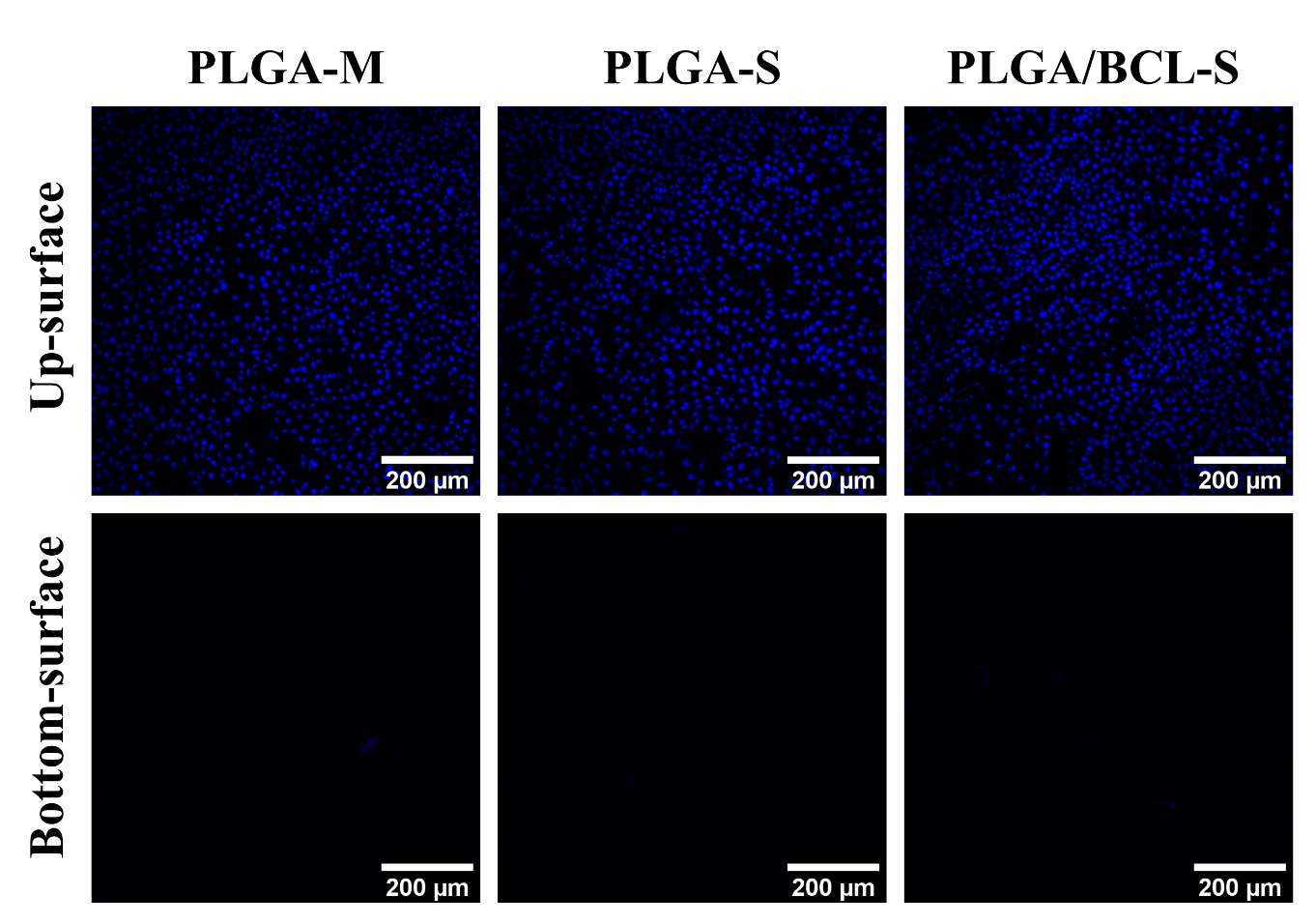


**Figure S4.** Fluorescence images of L929s cultured on the up-surfaces and penetrated cells on the bottom-surfaces of PLGA-M, PLGA-S, and PLGA/BCL-S after 3 days (blue for cell nucleus).


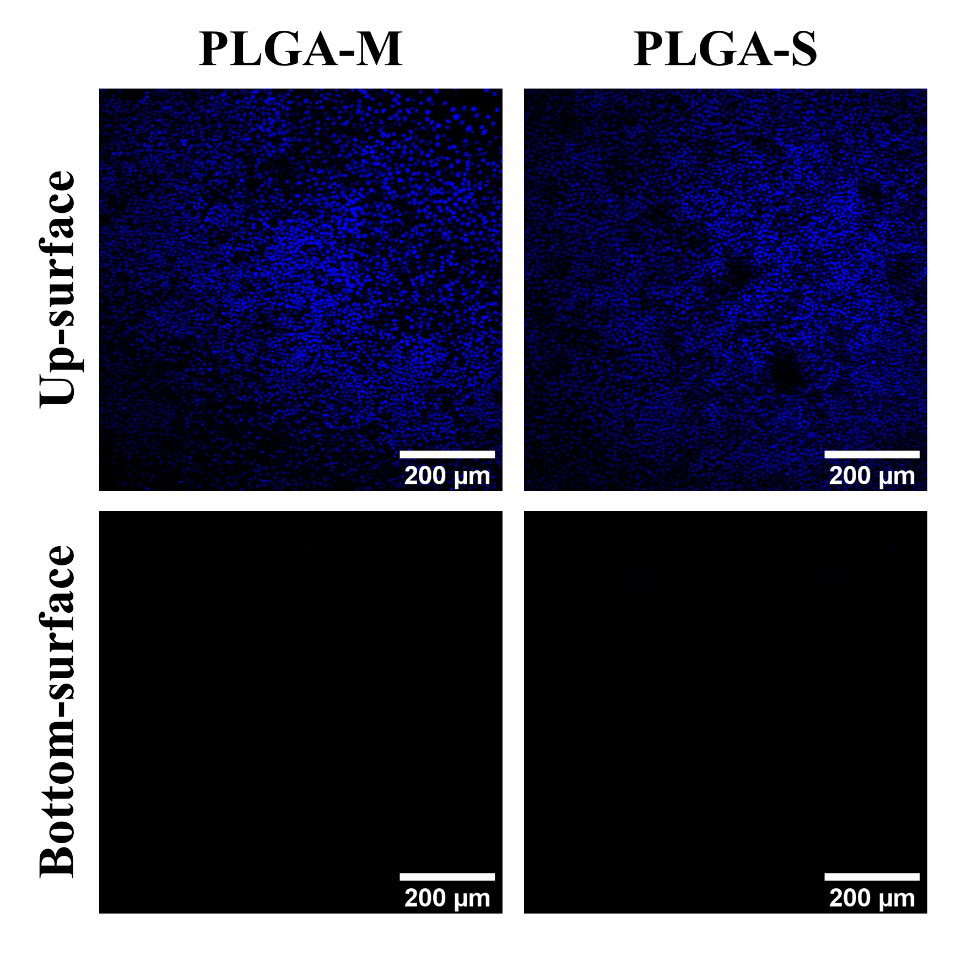


**Figure S5.** Fluorescence images of L929s cultured on the up-surfaces and penetrated cells on the bottom-surfaces of PLGA-M and PLGA-S after 7 days (blue for cell nucleus).


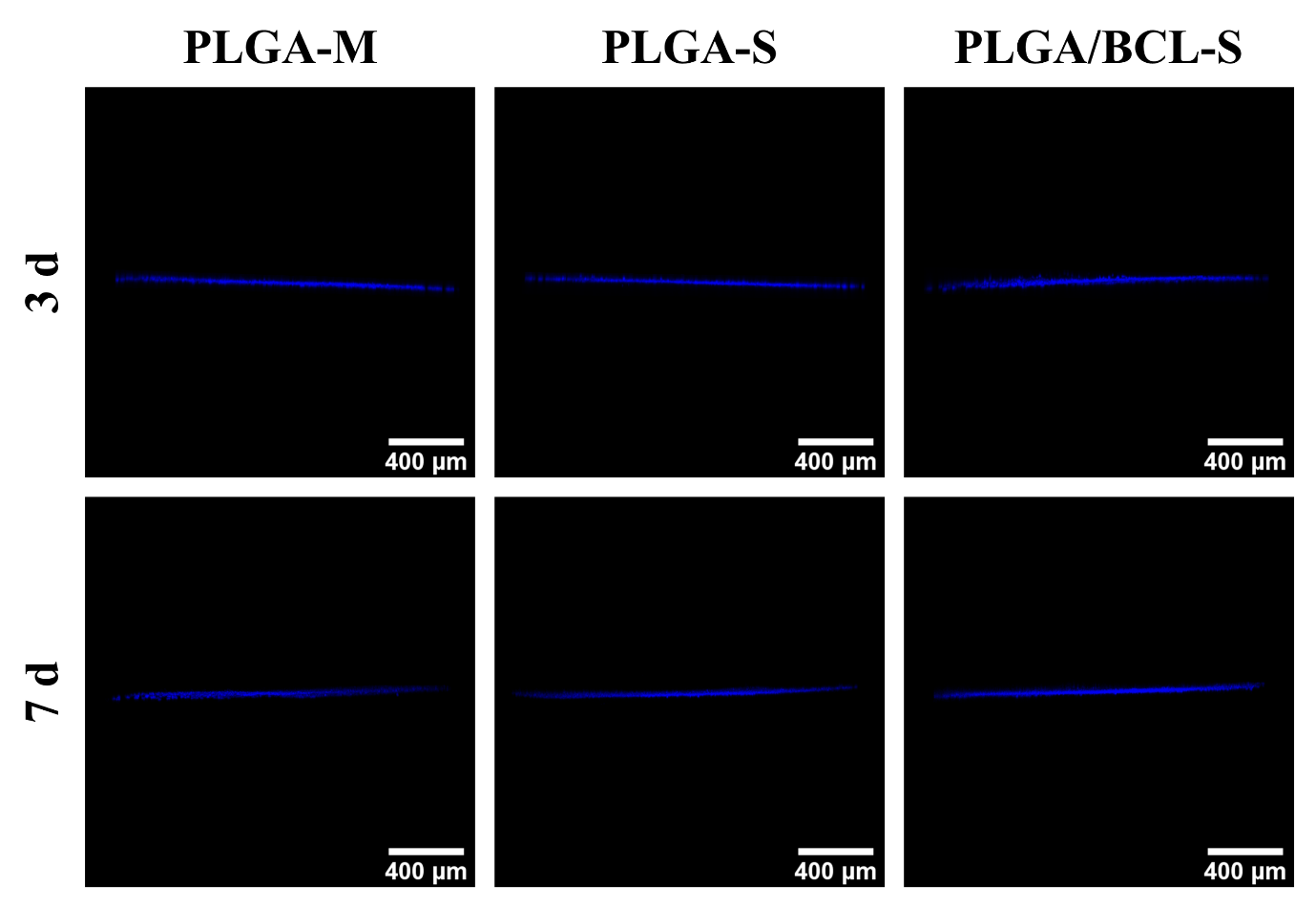


**Figure S6.** Cross-section images of penetration depth of L929s cultured on the up-surfaces of samples for 3 and 7 days (blue for cell nucleus).


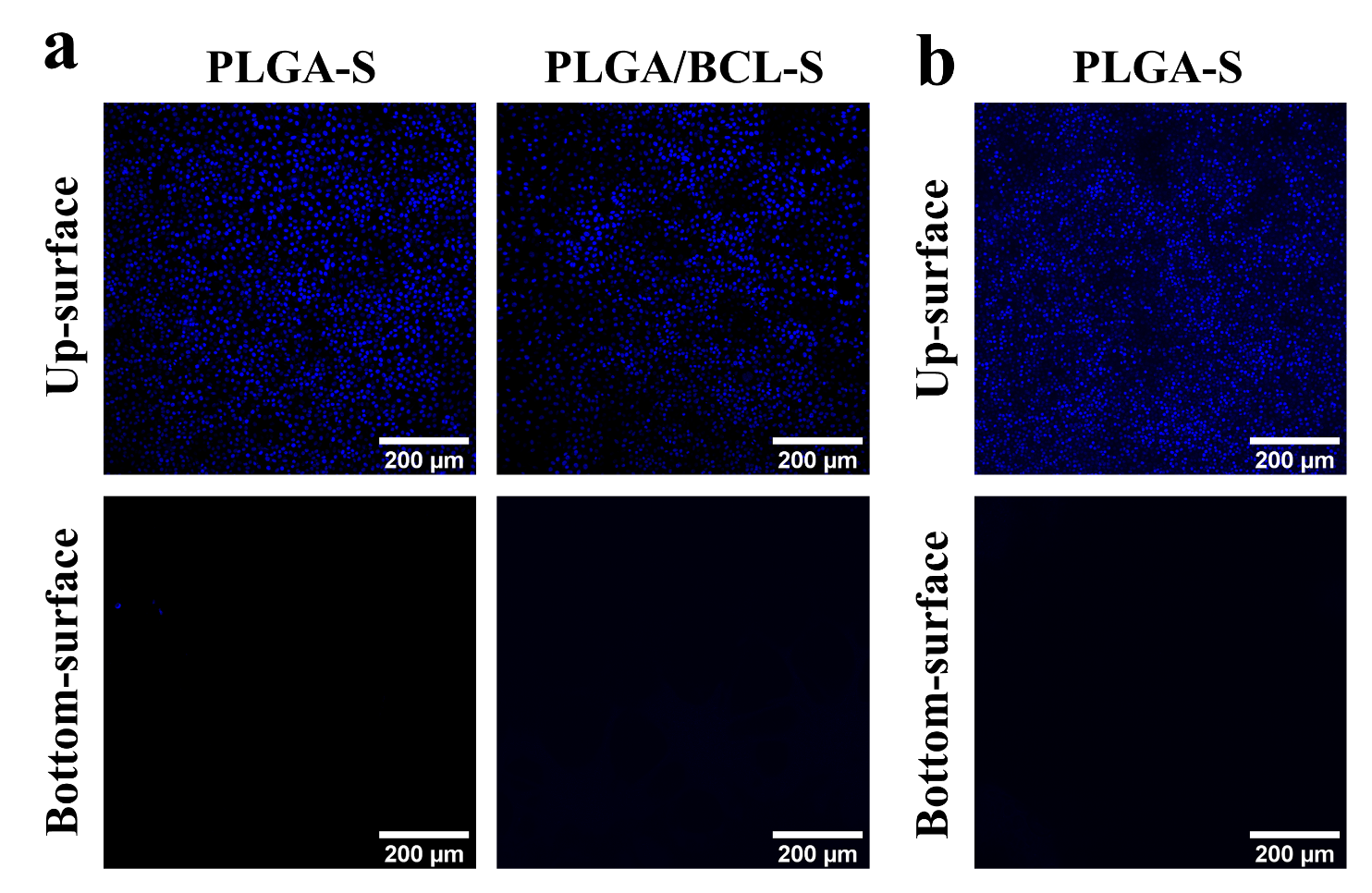


**Figure S7.** Fluorescence images of L929s cultured on the up-surfaces and penetrated cells on the bottom-surfaces of PLGA-S and PLGA/BCL-S after 3 days (a) and 7 days (b), exhibiting their barrier functions against fibroblasts after simulated clinical immersion for 4 weeks (blue for cell nucleus).


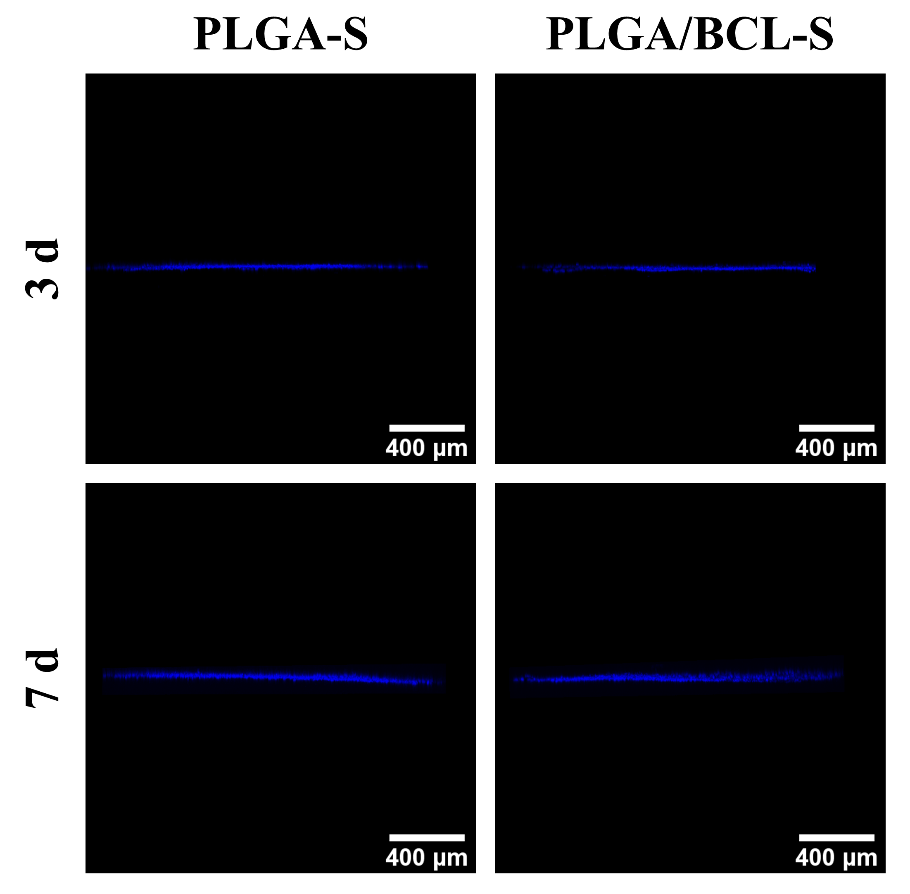


**Figure S8.** Cross-section images of penetration depth of L929s cultured on the up-surfaces of PLGA-S and PLGA/BCL-S for 3 and 7 days after simulated clinical immersion for 4 weeks (blue for cell nucleus).


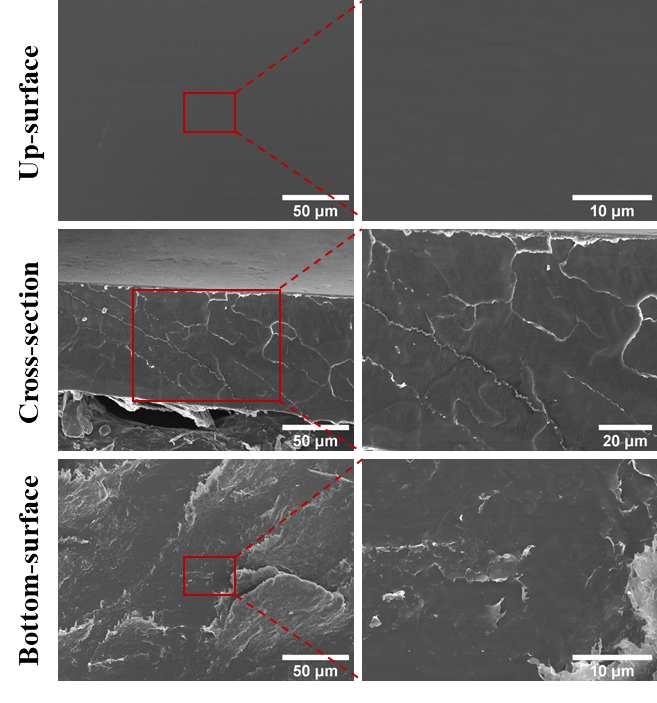


**Figure S9.** SEM images of relatively non-porous structure of PLGA-M.


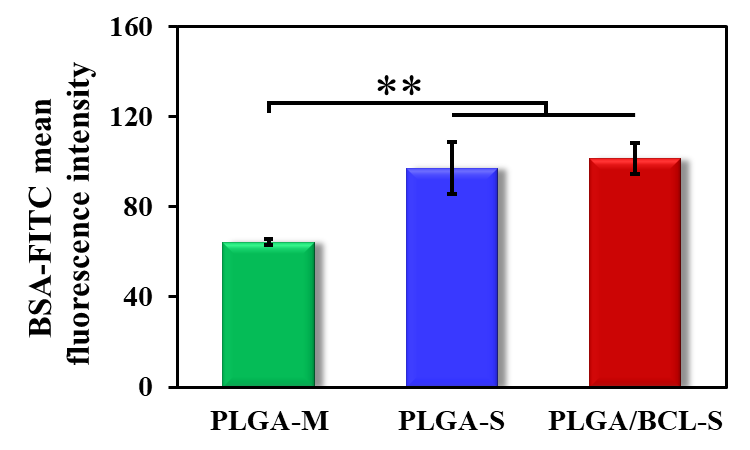


**Figure S10.** Semi-quantitative analyses of BSA adsorbed on the bottom-surfaces of PLGA-M, PLGA-S and PLGA/BCL-S (n = 3 independent samples; one-way ANOVA followed by LSD test; ** *p* < 0.01; error bars = SD).


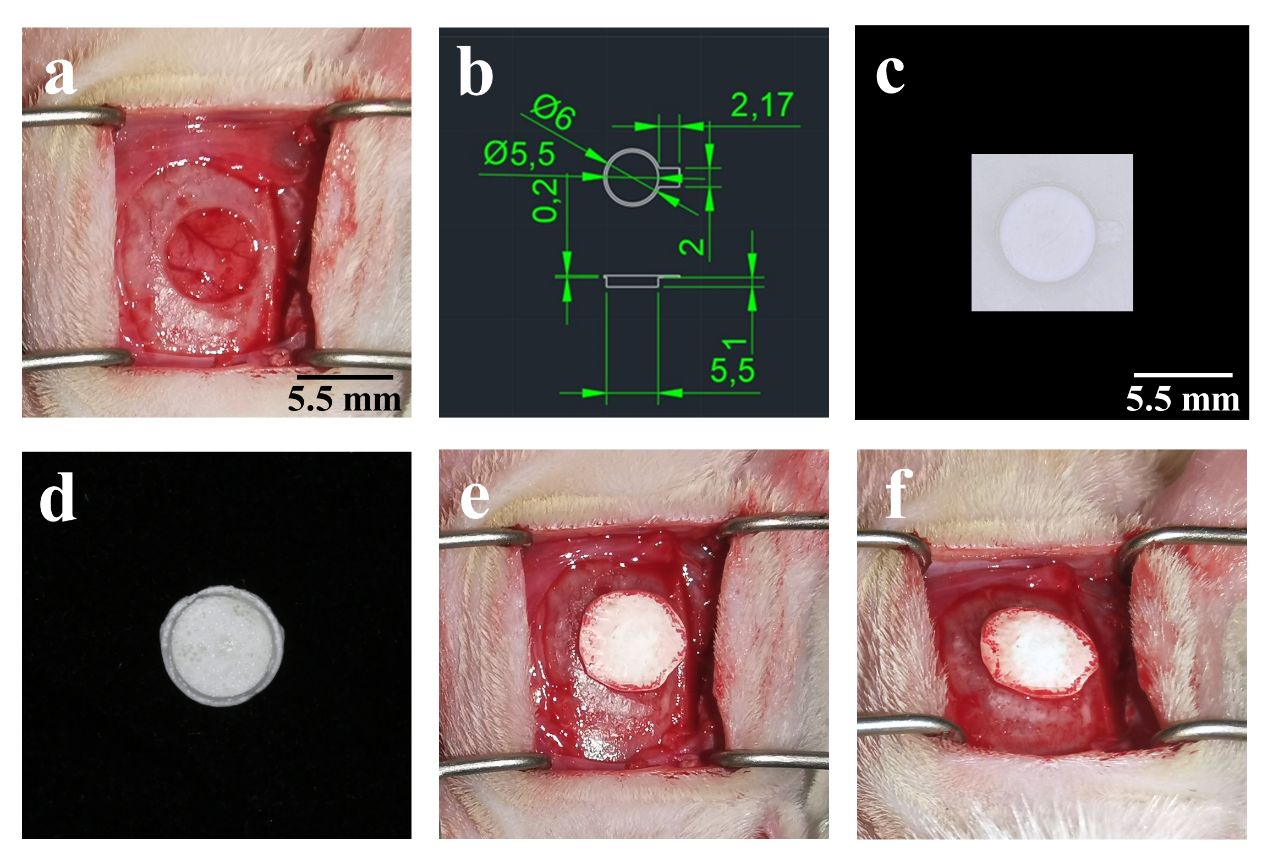


**Figure S11.** Surgical procedures of a rat calvarial defect model. a) Digital photo of a defect (5.5 mm diameter) created by a trephine bur. b,c) The negative mold of the target scaffold with a brim-like structure designed (b) and manufactured (c) via CAD/CAM technology. d) Digital photo of bottom-surface of PLGA/BCL-S. e,f) Digital photos of PLGA/BCL-S placed in bone defect region, exhibiting good matching of the scaffold with the bone defect.


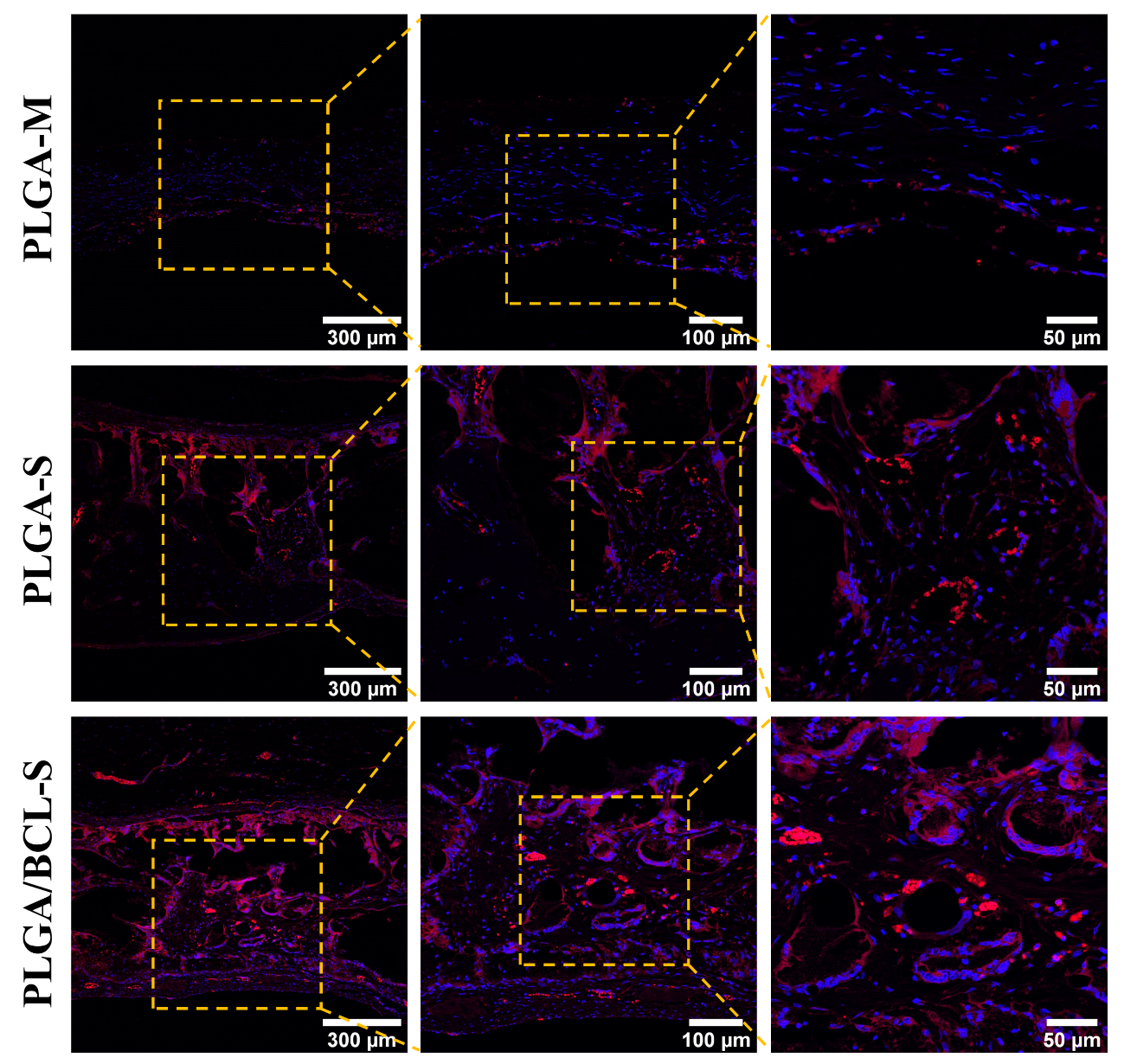


Figure S12. Images of IF staining of CD31 of calvarial decalcified sections after 8 weeks of implantation (blue for cell nucleus, red for CD31).

**Table S1. Primer sequences used for RT-qPCR**

| **Genes** | **Primers** |
| --- | --- |
| *GAPDH* | Forward: 5’ – TTGCCTTGCTGCTCTACCTCCA – 3’ |
|  | Reversed: 5’ – GATGGCAGTAGCTGCGCTGATA – 3’ |
| *VEGF* | Forward: 5’ – GTCTCCTCTGACTTCAACAGCG – 3’ |
|  | Reversed: 5’ – ACCACCCTGTTGCTGTAGCCAA – 3’ |
| *Ang-1* | Forward: 5’ – CCACACGTGGAACCGGATTT – 3’ |
|  | Reversed: 5’ – CGAGAAGTTTGATTTAGTACCTGGG – 3’ |
